# “Third Window” and “Single Window” Effects Impede Surgical Success: Analysis of Retrofenestral Otosclerosis Involving the Internal Auditory Canal or Round Window

**DOI:** 10.3390/jcm8081182

**Published:** 2019-08-07

**Authors:** Yun Jung Bae, Ye Ji Shim, Byung Se Choi, Jae-Hyoung Kim, Ja-Won Koo, Jae-Jin Song

**Affiliations:** 1 Department of Radiology, Seoul National University Bundang Hospital, 300 Gumi-dong, Bundang-gu, Seongnam 13620, Korea; 2 Department of Otorhinolaryngology-Head and Neck Surgery, Seoul National University, Hospital Healthcare System Gangnam Center, 152 Tehran Street, Gangnam-gu Seoul 06236, Korea; 3 Department of Otorhinolaryngology-Head and Neck Surgery, Seoul National University Bundang Hospital, 300 Gumi-dong, Bundang-gu, Seongnam 13620, Korea

**Keywords:** otosclerosis, cochlea, hearing, round window, stapes surgery

## Abstract

Background and Objectives: We aimed to identify prognostic computed tomography (CT) findings in retrofenestral otosclerosis, with particular attention paid to the role of otosclerotic lesion area in predicting post-stapedotomy outcome. Materials and Methods: We included 17 subjects (23 ears) with retrofenestral otosclerosis who underwent stapedotomy. On preoperative CT, the presence of cavitating lesion and involvement of various subsites (cochlea, round window [RW], vestibule, and semicircular canal) were assessed. Pre- and post-stapedotomy audiometric results were compared according to the CT findings. The surgical outcomes were analyzed using logistic regression with Firth correction. Results: Cavitating lesions were present in 15 of 23 ears (65.2%). Involvement of the RW was the strongest predictor of unsuccessful surgical outcome, followed by involvement of the internal auditory canal (IAC) and the cochlea. Conclusions: RW and IAC involvement in retrofenestral otosclerosis were shown to predict unsuccessful outcomes. While a “third window” effect caused by extension of a cavitating lesion into the IAC may dissipate sound energy and thus serve as a barrier to desirable postoperative audiological outcome, a “single window” effect due to an extension of retrofenestral otosclerosis into the RW may preclude a good surgical outcome, even after successful stapedotomy, due to less compressible cochlear fluid and thus decreased linear movement of the piston.

## 1. Introduction

Otosclerosis is a disorder of the otic capsule characterized by abnormal resorption of the endochondral bone and deposition of vascular bone, resulting in conductive, sensorineural, or mixed hearing loss [1,2]. This disorder can be classified into two types: (1) fenestral otosclerosis, which is confined to the lateral wall of the bony labyrinth, anterior to the oval window (OW) at the fissula ante fenestram; and (2) retrofenestral otosclerosis, with additional otosclerotic foci extending within the perilabyrinthine region [3,4,5,6,7]. 

Since Shea first reported surgical treatment of otosclerosis [8], stapes surgery has been established as the standard treatment, and shows good hearing outcomes and minimal complications [7,9,10]. However, it may not be possible to achieve sufficient air–bone gap closure (ABGC) in some cases, even after successful stapes surgery. Preoperative temporal bone computed tomography (TBCT) is a reliable method to detect otosclerotic lesions presenting as hypodense or radiolucent areas in the otic capsule [2,3,4,5,7,11,12]. In addition, TBCT has been shown to be useful for relating the distribution of otosclerotic lesions to audiometric results [6,7,10,11,13]. Previous studies have shown that retrofenestral otosclerosis is associated with poorer preoperative hearing thresholds [6,7,11,13,14] and post-stapedotomy audiological outcomes compared to fenestral otosclerosis [10,15,16]. In particular, our recent study showed that retrofenestral otosclerosis with a cavitating lesion involving the internal auditory canal (IAC) showed a poorer postoperative audiological outcome than those without any cavitating lesions; this was attributed to a “third window” effect that may act as a route for sound energy shunting and thus hamper the expected ABGC [17]. 

To our knowledge, however, there have been no studies to determine which retrofenestral subsites are critically related to poor post-stapes surgery hearing outcome. Although the involvement of the IAC was shown to be a poor prognostic factor in our previous study, other retrofenestral areas were not taken into account with regard to postoperative audiological outcome.

As a follow-up to our previous study [17], we further assessed preoperative TBCT to determine all possible anatomical predictors of post-stapedotomy hearing outcome in patients with retrofenestral otosclerosis. This study was performed to determine the prognostic value of TBCT findings in retrofenestral otosclerosis, and in particular to identify possible anatomical predictors of post-stapedotomy hearing outcome other than IAC involvement.

## 2. Materials and Methods

### 2.1. Subjects

The Institutional Review Board of the Clinical Research Institute of Seoul National University Bundang Hospital approved this retrospective study (IRB No. B-1810/499-104), and the requirement for informed consent was waived. Between March 2004 and August 2017, 65 patients were radiologically diagnosed with otosclerosis on TBCT. Of these patients, 31 with retrofenestral otosclerotic lesions on TBCT were initially recruited to the study, among whom those who did not undergo stapes surgery (*n* = 8), those undergoing cochlear implantation (CI; *n* = 4), and those for whom pre- and/or post-stapedotomy audiometric test results were not available (*n* = 2) were excluded. Therefore, the final study population consisted of 17 patients (7 males and 10 females; age range: 21–61 years; median age, 46.0 years) diagnosed with retrofenestral otosclerosis and treated with stapedotomy. Of these 17 patients, 6 had bilateral retrofenestral otosclerosis and were treated with bilateral stapedotomy. Therefore, a total of 23 operated ears were finally included in the analysis. Demographic data were collected based on the electronic medical records.

### 2.2. Temporal Bone Computed Tomography (TBCT) Protocol

TBCT was performed using a 64-section (*n* = 11) or 256-section (*n* = 6) multidetector CT scanner (Brilliance and iCT; Philips Healthcare, Best, The Netherlands). The imaging protocol involved helical acquisition at 120 kVp, 250 mAs, with a pitch of 0.825. Images were obtained at a slice thickness of 0.67 mm in increments of 0.33 mm and reformatted to a section thickness of 0.7 mm with no gaps. Both axial and coronal reformations were included in the image review.

### 2.3. Imaging Analysis

**Figure 1:**
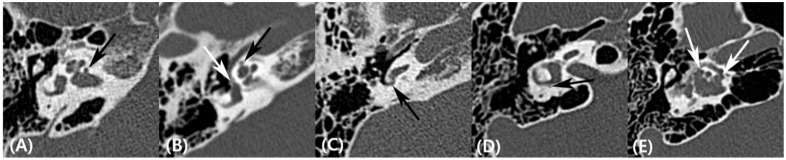
(**A**) Axial computed tomography (CT) image showing the presence of a focal hypodense notch connecting to the anterior wall of the internal auditory canal (IAC), designated as a cavitating lesion with IAC involvement (arrow). (**B**) Axial CT image showing otosclerotic involvement of the cochlear (black arrow) and the vestibule (white arrow). (**C**,**D**) Axial CT images showing retrofenestral involvement of the (**C**) round window and (**D**) the lateral semicircular canal (arrows). (**E**) Confluence between the cavitating lesion involving the IAC and cochlear involvement was demonstrated on axial CT (arrows).

Two board-certified neuroradiologists (Y.J.B. and B.S.C, with 9 and 19 years of experience, respectively), who were blinded to the clinical information, evaluated the results of TBCT by consensus. The subtype of otosclerosis was classified according to the previous criteria suggested by Veillon et al. [18]; Type 1A, restricted to the footplate; Type 1B, hypodense focus <1 mm, anterior to the oval window, at the level of the fissula ante fenestram; Type 2, hypodense focus >1 mm, anterior to the oval window, without contact with the cochlear endosteum; Type 3, hypodense focus >1 mm, anterior to the oval window, in contact with the cochlear endosteum; Type 4A, extensive hypodense foci located in the middle layer of the otic capsule, laterally, medially, and anterior to the cochlea; Type 4B, extensive hypodense foci around the semicircular canals (SCCs). On preoperative TBCT of each ear, the presence of a focal hypodense notch connecting to the anterior wall of the IAC was designated as a cavitating lesion involving the IAC (Figure 1A) [2,19,20,21]. In addition, the following retrofenestral subsites of otosclerotic involvement were assessed separately: cochlea, round window (RW), vestibule, and SCC (Figure 1B–D). In addition, the confluence between the cavitating lesion and cochlear involvement was recorded (Figure 1E).

### 2.4. Audiological Data Analysis

Pre- and post-stapedotomy pure tone audiograms were collected from all 17 patients before and 2 months after stapedotomy. Audiological data were collected as recommended by the guidelines of the Committee on Hearing and Equilibrium of the American Academy of Otolaryngology-Head and Neck Surgery (AAOHNS) [22]. The mean air conduction (AC) and bone conduction (BC) thresholds were averaged using 0.5, 1, 2, and 3 kHz thresholds [22,23,24,25,26,27]. The air–bone gap (ABG) and ABGC were calculated based on the mean AC and BC levels. A postoperative AC threshold lower than the preoperative BC threshold was referred to as overclosure [10]. Surgical success was defined as post-stapedotomy ABGC within 10 dB without BC deterioration [16]. Post-stapedotomy ABGC > 10 dB, or with BC deterioration after stapedotomy, was defined as surgical failure. 

### 2.5. Statistical Analysis

Continuous variables are expressed as means ± standard deviation. Based on the retrofenestral subsites, demographic data, mean AC, BC, ABG and ABGC were compared using the Mann–Whitney U test, Fisher’s exact test, and linear association. Surgical failure according to the retrofenestral subsites was compared using Fisher’s exact test and linear-by-linear association. We further utilized univariate logistic regression with Firth correction [28] to determine subsite involvements that could predict surgical failure. Statistical analyses were performed using SPSS (version 17.0; SPSS Inc., Chicago, IL, USA) and SAS software (version 9.3; SAS Institute, Cary, NC, USA). In all analyses, *p* < 0.05 was taken to indicate statistical significance.

## 3. Results

### 3.1. TBCT Findings

Out of total 65 subjects with otosclerosis, 26 subjects had cavitating lesions (40%); four out of 34 subjects with fenestral otosclerosis (11.8%) and 22 out of 31 subjects with retrofenestral otosclerosis (71%). The prevalence of the subtypes of the retrofenestral otosclerosis according to the CT classification was as follows; Type 3, 7.7%; Type 4A, 26.2%; Type 4B, 13.8%. Regarding the included 17 subjects with retrofenestral otosclerosis, data on the numbers of ears with otosclerotic lesions at each retrofenestral subsite are summarized in Table 1. Cavitating lesions involving the IAC were present in 15 of the 23 ears included in the study (65.2%). The cochlea was involved in 14 ears (60.9%), and the RW in 10 ears (43.5%). The vestibule was involved in all ears (100%), and SCC involvement was detected in eight ears (34.8%). Confluence between cavitating and cochlear lesions was found in six ears (26.1%).

### 3.2. Demographic and Audiological Results According to Retrofenestral Subsite

Table 2 presents the demographic characteristics and audiological results according to retrofenestral subsite.

The age of subjects did not differ significantly according to retrofenestral subsite involvement (*p* > 0.05). There was no significant difference between sexes in the involvement of any of the subsites, but cavitating lesions and confluence between cavitating and cochlear lesions were more prevalent in women than in men (*p* = 0.039 and *p* = 0.015, respectively).

Preoperative mean AC, BC, and ABG did not show significant differences according to retrofenestral subsite involvement (all *p* > 0.05). However, the presence of confluence between the cavitating and cochlear lesions was associated with significantly poorer preoperative mean AC and BC than in ears without confluence (*p* = 0.024 and *p* = 0.024, respectively). 

The ears with cavitating lesions involving the IAC showed poorer post-stapedotomy mean AC and BC than those without cavitating lesions (*p* = 0.004 and *p* = 0.011, respectively). Post-stapedotomy mean AC and BC were also significantly poorer in ears with versus without involvement of the cochlea (*p* = 0.033 and *p* = 0.016, respectively), RW (*p* = 0.002 and *p* = 0.012, respectively), and SCC (*p* = 0.047 and *p* = 0.013, respectively). Post-stapedotomy mean ABGs were significantly larger in ears with cavitating lesions (*p* = 0.028) and RW involvement (*p* = 0.005), but ABGC did not differ significantly according to the subsite involved (*p* > 0.05). Subjects with confluence between cavitating and cochlear lesions showed significantly poorer post-stapedotomy BC (*p* = 0.044) compared to those without such confluence, but there were no significant differences with regard to postoperative AC, ABG, or ABGC in relation to the presence or absence of confluence (all, *p* > 0.05). 

Six ears showed overclosure with a mean of 3.9 dB. The number of overclosures was significantly higher in ears with cavitating lesions (*p* = 0.042) and RW involvement (*p* = 0.001) than in those without involvement of the IAC or RW.

### 3.3. Correlation between Surgical Failure and Retrofenestral Subsites

Surgical success and failure rates after stapedotomy according to retrofenestral subsite are summarized in Table 3. The surgical failure rate was significantly higher in ears with cavitating lesions (*p* = 0.009), cochlear involvement (*p* = 0.036), and RW involvement (*p* = 0.003). As shown in Table 4, in univariate logistic regression analysis, cavitating lesion, cochlear involvement, and RW involvement were significant predictors of surgical failure. RW involvement was the strongest predictor of a poor prognosis (odds ratio (OR), 19.00; *p* = 0.006), followed by cavitating lesion (OR, 12.78; *p* = 0.016) and cochlear involvement (OR, 7.00; *p* = 0.035).

## 4. Discussion

TBCT has been reported to be effective for relating pre- and post-stapedotomy audiometric results with the distribution and extent of otosclerotic changes [6,7,10,11,13,14,15]. However, for predicting post-stapedotomy hearing outcome, TBCT has mainly been used to compare fenestral and retrofenestral otosclerosis [10,15,16]. For example, Lagleyre et al. [10] reported that postoperative BC was significantly lower in ears with pericochlear or endosteal involvement than in ears with isolated fenestral involvement. However, they did not compare hearing outcomes among retrofenestral otosclerosis patients with different sites of endosteal involvement. Marx et al. [15] obtained the same results by comparing extensive otosclerosis with isolated fenestral otosclerosis, but no comparisons were performed according to retrofenestral subsite. Moreover, Min et al. [29] reported contradictory findings wherein cochlear involvement did not affect the postoperative audiometric results, although their study population included only four subjects with cochlear involvement.

In our recent study, we demonstrated that patients with cochlear otosclerosis involving the IAC showed significantly poorer postoperative audiological outcome than those without any cavitating lesions [17]. With regard to that result, we proposed that otosclerotic foci extending to the IAC may have acted as a third window providing a route for sound energy, such that the expected ABG closure could not be achieved in some subjects with cavitating otosclerosis. However, that study merely compared cochlear otosclerosis subjects with and without IAC involvement, and much remained to be determined about the retrofenestral sites of involvement with regard to audiological outcome after stapedotomy. As a follow up to our recent study, we further assessed preoperative TBCT to determine all possible anatomical predictors of post-stapedotomy hearing outcome in patients with retrofenestral otosclerosis. The present study showed that post-stapedotomy AC and BC in the operated ears were significantly poorer in subjects with cavitating lesions (involvement of the IAC) or with involvement of the cochlea, RW, or SCC. The post-stapedotomy ABG was significantly larger in subjects with cavitating lesions or RW involvement than in subjects without involvement of these areas. RW involvement was found to be the strongest predictor of surgical failure, followed by the presence of a cavitating lesion and cochlear involvement.

### 4.1. Importance of Excavating Anatomical Subsites of Otosclerosis relevant to Surgical Outcome

The most common clinical presentation of otosclerosis is the fenestral type, resulting from osteodystrophic changes around the OW and fixation of the stapes footplate, and leading to conductive hearing loss [3,5,9,11]. In addition, sensorineural hearing loss up to profound hearing loss can develop in patients with retrofenestral otosclerosis [4,6,30]. The conventional treatment option for profound hearing loss due to advanced otosclerosis is CI [31,32,33]. However, stapedotomy with a hearing aid can also lead to a good outcome in some cases of advanced otosclerosis [34,35]. Therefore, prediction of stapedotomy outcome in patients with advanced retrofenestral otosclerosis is critical when selecting treatment options. In addition, for subjects with less severe conductive or mixed hearing loss, preoperative prediction of audiological outcome after stapedotomy may encourage realistic expectations regarding audiological outcome. Therefore, identification of radiological predictors of surgical outcome may be of great importance to patients with retrofenestral otosclerosis. 

Along with the previously suggested negative prognostic effect of advanced cochlear otosclerosis, we noted negative prognostic effects of the involvement of the IAC or RW [4,11,13,15]. Here, we focus on possible explanations for the status of IAC and RW involvement as negative prognostic factors with regard to post-stapedotomy hearing outcome.

### 4.2. Cavitating Otosclerosis Involving the IAC: A Third Window Affecting the Surgical Outcome

Cavitating lesions have been characterized in small case reports as a cause of third window or endosteal otosclerotic foci in combination with retrofenestral otosclerosis, but their clinical significance and relationship to otosclerosis have not been clarified [19,20,21]. Recently, Pippin et al. [2] assessed cavitating lesions classified as “IAC diverticula” in 810 TBCT scans of 807 patients, and found cavitating lesions combined with classic otosclerosis findings in 1% of the patients. They also surmised that cavitating lesions were potentially responsible for sensorineural hearing loss. In our previous study, cochlear otosclerosis with cavitating lesions involving the IAC showed significantly poorer audiological outcomes than those without any cavitating lesions.

In the present study, we detected cavitating lesions in 15 of 23 ears with retrofenestral otosclerosis (65.2%), which was a much higher prevalence rate than reported previously [2]. We assumed that this rate of cavitating lesions in ears with retrofenestral otosclerosis was higher than would be seen in ears with fenestral otosclerosis or the normal population, although the high rate may also have been due to the relatively small sample size in our study. In addition, the subtypes of 3, 4A and 4B was higher in our study than previous report [18]. We believe that, since our institution is a nation-wide third referral hospital, more advanced-stage patients might have been concentrated. 

**Figure 2:**
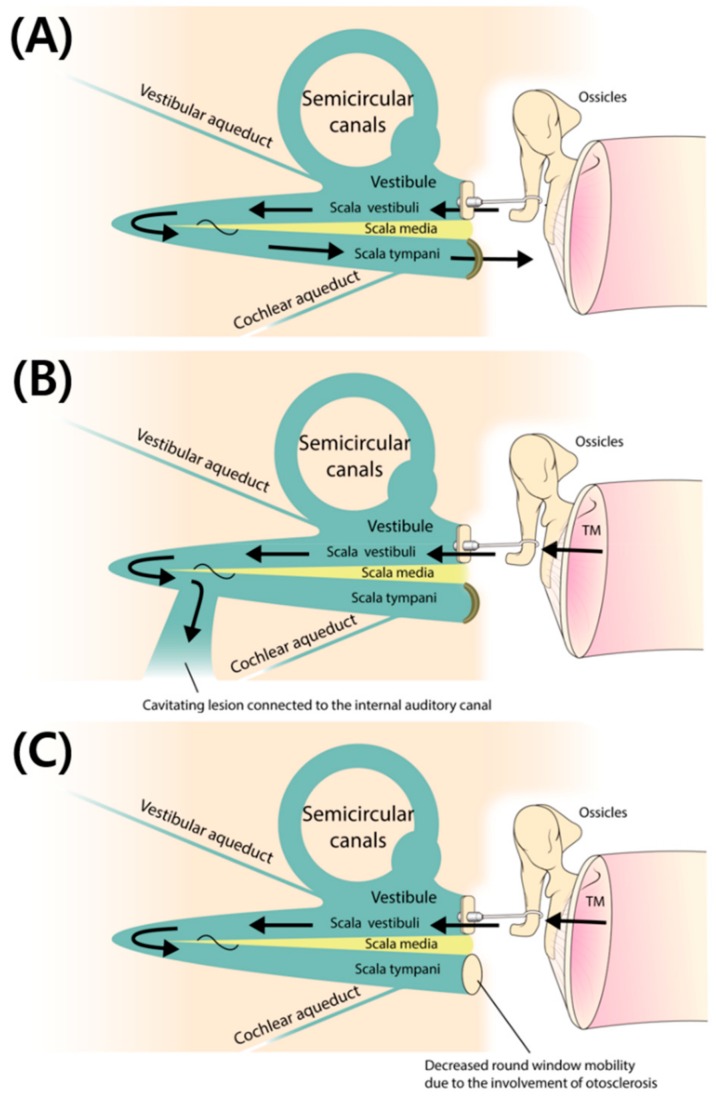
(**A**) Restoration of air conduction (AC) energy and near-normalized sound conduction in a subject with otosclerosis after stapedotomy. (**B**) Even after restoration of AC energy at the level of the footplate of the stapes by successful stapedotomy, shunting across the cavitating lesion to the internal auditory canal may play a role in dissipating sound energy, thus serving as a barrier to desirable postoperative audiological outcomes. (**C**) Decreased compliance of the round window due to otosclerosis involvement may result in increased mechanical load on the footplate, reduced volume velocity of the stapes, and thus decreased ossicular coupling presenting as increased air-bone gap. Adapted with permission from Rosowski, J.J. Conductive hearing loss caused by third window lesions of the inner ear [41].

Consistent with our recent findings [17], the post-stapedotomy AC, BC, and ABG were significantly poorer in ears with cavitating lesions involving the IAC than in ears with retrofenestral otosclerosis without IAC involvement (Table 2). In addition, the involvement of the IAC was the second strongest predictor of surgical failure (Table 3 and Table 4). That is, the presence of IAC involvement was again confirmed to be a significant negative prognostic factor with respect to surgical outcome in subjects with retrofenestral otosclerosis, regardless of cochlear involvement. Pericochlear cavitation extending to the IAC may have acted as a newly developed third window in these patients. As a result, even after restoration of AC energy at the level of the footplate of the stapes by successful stapedotomy, shunting across the cavitating lesion to the IAC may have played a role in dissipating sound energy, thus serving as a barrier to desirable postoperative audiological outcomes (Figure 2A,B).

### 4.3. Involvement of the Round Window (RW) in Subjects with Retrofenestral Otosclerosis: An Indicator of the “Single Window” Effect 

The RW is the second most common site of involvement in otosclerosis, after the fissula ante fenestram [36]. Although involvement of the RW is a definite indicator of retrofenestral otosclerosis, it should not be regarded as an indicator of advanced otosclerosis, where in a previous study about 35% of subjects with RW involvement did not show pericochlear extension of otosclerosis, and there were even patients with isolated RW otosclerosis [36].

As summarized in Table 2, the post-stapedotomy AC, BC, and ABG were significantly poorer in ears with involvement of the RW than in ears without RW involvement. Moreover, univariate logistic regression analysis with Firth correction showed that RW involvement was the strongest predictor of surgical failure (Table 3 and Table 4). That is, the involvement of the RW in subjects with retrofenestral otosclerosis was the strongest indicator of a poor post-stapedotomy audiological outcome. These results were consistent with previous reports indicating that RW involvement is responsible for residual or conductive hearing loss, even after successful stapedectomy, in about one quarter of cases undergoing revision surgery [10,37].

Previous reports have suggested that increased impedance of the perilymph and decreased vibratory motion of the basilar membrane may hamper the compression mechanism of BC and thus result in an increased BC threshold [36,38]. In addition, decreased compliance of the RW due to otosclerosis involvement may result in increased mechanical load on the footplate, reduced volume velocity of the stapes, and thus decreased ossicular coupling presenting as increased ABG [36,39]. That is, decreased interplay between the OW and RW may result in an increase in the ABG [40]. Considering these changes in subjects with RW involvement, successful stapedotomy cannot always guarantee successful ABGC, because the cochlear fluid is less compressible and the inserted piston may exhibit decreased linear movement [36,39]. With reference to the third window effect of IAC involvement, we have coined the term “single window” pertaining to the effect of RW involvement (Figure 2C).

### 4.4. Limitations of This Study and Proposals for Future Investigations

Although we identified significant post-stapedotomy outcome predictors based on preoperative radiological findings, the present study had several limitations. First, due to the relatively small number of subjects included in the study, we could not perform multivariate logistic regression analysis [42]. Although we minimized the effect of this limitation by adopting univariate logistic regression analysis with Firth correction [28], further studies including larger numbers of subjects are required to generalize our preliminary findings. Second, we did not quantitatively assess the effect of the size or density of the otosclerotic foci (on CT) on the hearing results, where these parameters may have acted as confounding factors. There were a number of reasons why we did not perform these measurements. First, there is still controversy regarding whether the size or density of otosclerotic lesions can actually have an impact on hearing threshold [11,13,14,15,29]. Second, measurement of otosclerotic lesions on CT may not be reliable, because accuracy with respect to defining the boundaries of the lesion can be limited by the current spatial resolution. Instead, we assessed confluence between cavitating and cochlear lesions, the presence of which could be indicative of a large lesion. Third, this was a retrospective chart review including a relatively small number of subjects with otosclerosis, drawn from a single center. To generalize our preliminary findings, further studies in larger numbers of subjects with retrofenestral otosclerosis are required.

## 5. Conclusions

Taken together with previous reports regarding pericochlear endosteal involvement, RW and IAC involvement in retrofenestral otosclerosis were shown to be predictors of unsuccessful surgical outcomes. Along with the third window effect (i.e., extension of a cavitating lesion to the IAC), a single window effect due to extension of retrofenestral otosclerosis to the RW may preclude a good surgical outcome, even after successful stapedotomy. Therefore, precise identification of cavitating lesions on preoperative TBCT is mandatory for appropriate evaluation and counseling of subjects with retrofenestral otosclerosis.

## Figures and Tables

**Table 1:** Numbers of the ears with otosclerotic foci at each anatomical subsite.

Subsite	No.
Cavitating lesion	15
Cochlea	14
RW	10
Vestibule	23
SCC	8
Confluence between cavitating lesion and cochlear involvement	6

**Table 2:** Demographic and Audiometric Results according to the Retrofenestral Subsites.

Demographic and Audiometric Data	Cavitating lesion			Cochlea			RW			SCC			Confluence		
	Yes	No	*P*	Yes	No	*P*	Yes	No	*P*	Yes	No	*P*	Yes	No	*P*
Age (years)	46.9 ± 10.3	47.3 ± 5.0	0.825	45.6 ± 9.85	49.1 ± 6.5	0.224	48.7 ± 7.8	45.7 ± 9.5	0.879	49.3 ± 12.8	45.8 ± 5.7	0.131	47.7 ± 6.5	45.0 ± 13.8	0.708
Sex (n, female/male)	11/4	2/6	0.039 *	9/5	4/5	0.417	8/2	5/8	0.09	6/2	7/8	0.379	6/0	7/10	0.015 *
Pre-stapedotomy AC (dB)	59.8 ± 18.9	55.3 ± 8.8	0.925	61.8 ± 18.5	52.6 ± 9.8	0.305	64.8 ± 20.3	53.2 ± 10.0	0.376	65.5 ± 24.2	54.3 ± 8.0	0.466	72.9 ± 24.0	53.0 ± 8.0	0.024 *
Post-stapedotomy AC (dB)	44.4 ± 16.4	24.7 ± 7.4	0.004 *	43.5 ± 15.6	28.3 ± 14.7	0.033 *	49.9 ± 15.1	28.1 ± 10.9	0.002 *	47.2 ± 19.7	32.4 ± 12.8	0.047 *	46.5 ± 19.6	34.4 ± 15.0	0.155
Pre-stapedotomy BC (dB)	34.2 ± 13.4	28.9 ± 8.3	0.728	34.9 ± 13.4	28.3 ± 8.5	0.277	36.9 ± 14.1	28.8 ± 9.0	0.284	38.8 ± 15.3	28.9 ± 8.4	0.131	44.4 ± 15.7	29.1 ± 6.7	0.024 *
Post-stapedotomy BC (dB)	31.5 ± 12.5	19.2 ± 5.2	0.011 *	31.7 ± 12.4	20.1 ± 7.1	0.016 *	34.6 ± 13.3	21.5 ± 7.1	0.012 *	36.2 ± 14.5	22.4 ± 7.0	0.013 *	36.8 ± 15.4	23.8 ± 8.7	0.044 *
Pre-stapedotomy ABG (dB)	25.6 ± 8.6	26.4 ± 8.9	0.681	26.9 ± 8.3	24.3 ± 9.1	0.643	27.9 ± 8.0	24.3 ± 8.9	0.376	26.7 ± 9.7	25.4 ± 8.2	0.975	28.5 ± 10.5	24.9 ± 7.8	0.609
Post-stapedotomy ABG (dB)	13.0 ± 10.1	5.5 ± 4.3	0.028 *	11.8 ± 7.2	8.2 ± 11.7	0.053	15.3 ± 10.1	6.5 ± 6.4	0.005 *	11.0 ± 7.0	10.0 ± 10.3	0.428	9.7 ± 6.5	10.6 ± 10.1	0.812
ABGC (dB)	12.6 ± 12.3	20.9 ± 7.8	0.065	15.1 ± 8.3	16.1 ± 15.8	0.516	12.6 ± 14.5	17.8 ± 8.4	0.483	15.7 ± 9.0	15.4 ± 12.9	0.875	18.8 ± 7.8	14.3 ± 12.5	0.392

* *p* values less than 0.05; RW, round window; SCC, semicircular canal; Confluence, confluence between cavitating lesion and cochlear involvement; AC, air conduction; BC, bone conduction; ABG, air bone gap; ABGC, air bone gap closure.

**Table 3:** Surgical success and failure according to the retrofenestral subsites.

Subsite	Surgical Success	Surgical Failure	*p*-Values
Cavitating lesion (n, Yes/No)	4/7	11/1	0.009 *
Cochlea (n, Yes/No)	4/7	10/2	0.036 *
RW (n, Yes/No)	1/10	9/3	0.003 *
SCC (n, Yes/No)	2/9	6/6	0.193
Confluence between cavitating lesion and cochlear involvement (n, Yes/No)	2/9	4/8	0.365

* *p*-values less than 0.05; RW, round window; SCC, semicircular canal.

**Table 4:** Univariate logistic regression analysis to determine subsites predicting surgical failure.

Subsite	Odds Ratio	95% CI	*p*-Values
Cavitating lesion	12.78	1.62–100.7	0.016 *
Cochlea	7	1.15–42.7	0.035 *
RW	19	2.32–155.9	0.006 *

* *p*-values less than 0.05; CI, confidence interval; RW, round window.
